# Trotter welfare’s protection: A legislative perspective

**DOI:** 10.14202/vetworld.2015.427-431

**Published:** 2015-03-30

**Authors:** Annamaria Passantino, Claudia Giannetto, Letizia Passantino, Giuseppe Piccione

**Affiliations:** 1 Department of Veterinary Sciences, University of Messina, Polo Universitario dell’Annunziata, 98168, Messina, Italy; 2 Department of Emergency and Organ Transplant (D.E.T.O.), University of Bari, Str. Prov.le per Casamassima Km 3, 70010 Valenzano (BA), Italy

**Keywords:** animal protection, animal welfare, five freedoms, guidelines, legislation, trotter horses

## Abstract

The Council of Europe’s activities in the field of animal welfare are particularly noteworthy and comprise the elaboration of several norms for the protection of animals. Concerning the specific European Directive, Regulations or Convention for the protection of animals, the Authors underline the missing of specifics recommendations concerning the welfare of sport horses and especially of trotters. Guidelines are reported by regulation of equestrian sports. The paper’s purpose is to give practical elements to individuate the welfare state and to promote a clear regulation on welfare, care and protection of trotters.

## Introduction

In the last century, the European Union Member perceived the need to safeguard animals and their welfare. The stages of this evolution are marked by three main documents. Starting from the Universal Declaration of Animals’ Rights (15^th^ October 1978), animals acquired their rights and violating them humans commit a crime against the nature. In 1987, the European Convention for the Protection of Companion Pet Animals (1^3th^ November 1987) gave a radical change of perspective in the juridical guardianship due to the awareness that animal was a psycho-physical entity, capable of experiencing feelings and emotions, pain and anguish, a subject with rights that has to be safeguarded, like man does [1-4]. The recognition of animal dignity as sentient beings is contained in the Protocol on Animal Protection and Welfare, attached to the final act of the institutive Treaty of the European Union, approved in 1997 in Amsterdam and then in 2007 in Lisbon [5,6]. Little research has investigated the domestic horses’ welfare, and most knowledge is based on empirical findings rather than on scientific evidence [7].

Partnership between men and horses has a long history; in fact, horses were domesticated by humans more than 4000 years ago and have played a central role in the process of civilization [8]. Horses are social animals that live in groups in close contact with conspecifics, and they spend most of the day hours walking and grazing. Some of the constraints imposed on horses during the last century are in conflict with their natural behavior. This is in contrast to the ethical concept that should prevent man from abusing and using animals for personal entertainment or earnings.

In the second half of the twentieth century, equestrian sports became very popular, the standard of competition improved dramatically, and nowadays, horses competing in international competitions are expected to perform at a very high level. Therefore, they have to be trained and managed in a way that leads to an appropriate physical fitness level according to the kind of competition [8].

Many horses do not achieve their full athletic potential as a consequence of inappropriate methods of conditioning, training or management that can lead to injuries or poor welfare conditions [7].

Although sport animal welfare has long been recognized as part of applied ethology, but how welfare affects horses performance has received poor attention. For this reason, equine clinicians have to make an ethical code in order to protect athletic horses from equine industry that often does not consider the biological needs of horses itself. At this purpose, veterinarians have the scientific knowledge that allows them to assess animal welfare.

In this paper, the authors: (a) Deal with the lack of specific laws and/or regulations with regard to sport horse (especially trotters) welfare and (b) suggest guidelines in order to promote high standards of trotter welfare trough the assessment of elements indicators of good welfare.

## Horse Welfare

In recent years, animal welfare has been the subject of intense debate and public concern. The terms welfare and protection are often used as synonyms. The word protection means moral right, and it includes the protection of animals as well as its physiological, emotional and psychological point of view [9]. The term welfare requires strict definition if it is to be used effectively and consistently. A clearly defined concept of welfare is needed in precise scientific measurements, legal documents and public statements or debates. If animal welfare is to be compared in different situations or evaluated in specific situations, it must be assessed in an objective way.

The assessment of animal welfare aside from any Ethical Judgment has to provide objective information about animal conditions.

A suitable definition of animal welfare must refer to intrinsic characteristics of the animal rather than to something concedes by man.

In fact, an animal is in a good state of welfare (as indicated by OIE and set out in Chapter 7.1. Introduction to the recommendations for animal welfare, art. 7.1.1. of the OIE Terrestrial Animal Health Code) when it is healthy, comfortable, well nourished, safe, able to express innate behavior, and without suffering from unpleasant states such as pain, fear and distress [10].

In order to sum up the concept of welfare it must be viewed from a scientific point of view, where the physiological and psychological results of environmental stress, and the impact of it on an organism, are measured upon the base of a scale ranking with ‘very good’ at one end and ‘very poor’ at the other.

The concept of animal welfare is based on the respect of five fundamental principles (freedom) as stated in 1992 during the Farm Animal Welfare Council. These principles are: (a) Freedom from thirst, hunger and malnutrition; (b) freedom from discomfort; (c) freedom from pain, injury, disease; (d) freedom to express normal patterns of behavior; (e) freedom from fear and distress [11].

These five freedoms can be incorporated in a tridimensional vision of animal welfare considering: naturalness (normal behavior), physical and emotional (or psychological) well-being [12]. These elements are strictly correlated to each other and could be at risk in trotter horse.

## Current Legislation

Since ancient time, horses have been used in farming and transport, but also as food source. However, nowadays, they are considered as companion animals used for recreational and social purposes (sport, competition, pet therapy). In all of these cases, the animals are subject to the owners care, use and treatment. Owners need to be aware of proper care principles to ensure that the welfare of the horse is not being sacrificed. The horses have specific needs, which should be fulfilled to have a proper welfare. The simple definition of horse welfare is not clear, and varied regions or cultures do not always agree on what is acceptable use versus an unacceptable welfare problem.

Over the last few decades, countries from all regions in the world have begun to promulgate legislation for minimum standards of animal protection. A variety of laws regulates several aspects of the welfare of domestic animals used for various purposes.

Legislation about horses deals with (a) transport, (b) veterinary medicinal products, (c) identification and (d) maltreatment.

The frequency, distance and method of transport to which the horse is subjected are largely dependent on the use to which a horse is put. Competition, breeding, leisure activities, sale or slaughter are common uses for transported horses. The characteristics and procedures of horses’ transportation mainly depend on horses use.

National and international trade and competitions involving athletic horses are becoming more frequent, and there is a need to limit potential sources of stress during transport to ensure adequate performances as well as to prevent poor welfare. The methods of horse transport have been reviewed by Houpt and Lieb [13], Leadon [14] and Lindner [15], with special emphasis on athletic horses.

The European Regulation (EC) No. 1/2005 [16] aims to improve animal welfare by extending transportation standards concerning vertebrates with economic activity [17,18]. It will apply to all those who are involved with transportation of live vertebrate animals in connection with an economic activity. This regulation does not define what “economic activity” means, however gives an indication that it includes transport for commercial purposes where an exchange of goods, money or services takes place and also where transport directly or indirectly involves or aims at financial gain. It does not include individual horses, fit for travel, transported by their owner or another responsible person. Transporting competition or pleasure horses and ponies is not regulated by transport legislation. Law on Veterinary Medicinal Products [19] implements the Community code relating to veterinary medicinal products. The European Regulation (EC) No. 122/2013 establishing a list of substances considered essential for the treatment of equid was published by the European Commission on 13 February 2013 and came into force on 16 February. It directly applies throughout the EU and therefore in Italy. Substances included in the annex of this Regulation can be administered to horses under a prescription from a veterinarian only if there is no suitable authorized product available. These substances can be used in the prescribing cascade, according to the clinical judgment of the prescribing veterinarian.

The details relating to identification of equid are contained within specific law [20] that set standards for the way horses must be microchipped and how the information is recorded. Furthermore it’s important underline that among various forms of violence and cruelty to all animal is included the administration of narcotics or prohibited substances or medication that harms their health and this fact is punished by the Law on Maltreatment of animals [1,21]. It must be also considered that specific regulations and ethical codes regarding sport horses management, without any legal value, have been produced by several associations and federations as: International Equestrian Federation Veterinary Regulation (2^nd^ Edition, effective 1 January 2015) [22], a code of conduct for the welfare of the horse destined to all the people involved in the international equestrian sport. This code requires all those people to acknowledge and accept that all times the welfare of the horse must be paramount and must never be subordinated to competitive or commercial influences.

In some countries, there are guidelines covering horses’ welfare. Especially, in Great Britain, where the equine are used primarily for sport and recreation, the horses’ needs are clearly explained in Code of Practice for the welfare of horses, ponies, donkeys and their hybrids [23]. The Animal Welfare Act 2006 [24] requires ensuring that any horses, ponies, donkeys or mules have a suitable environment to live in; has a healthy diet; can behave normally; has appropriate company; and is protected from pain, suffering, injury and disease. On the basis of these concepts, the equine industry has launched a health and welfare strategy for horses to advise people on the correct treatment of the animals.

### General principles for the welfare of trotters Proposals

In order to develop guidelines on how trotters should be kept, housed and used in respect of their welfare conditions, it is appropriate to determine what elements or standards should be met. In ours proposals, the words shall, must and should have the follow meanings: Shall show there is a statutory requirement; must indicate a minimum standard; should denote a strong recommendation.

In order to analyze and put the guidelines on the welfare of the horse trotter the value of the indicators of good welfare must always keep in mind. In particular, the parameters consider in the study of animal welfare can be direct and indirect [25]. The first ones concern animal management including stabling, feeding and training conditions. The second ones include elements that can be directly observed on the animal and, so, detectable even at work without having to be present in the location of housing.

## Direct Parameters

Among direct parameters stables have great importance; these must be safe, hygienic, comfortable, well ventilated and of sufficient size for the type and disposition of the horse. Recommendations on minimal box sizes may be found in several countries like Scotland or Germany. In a recent study, Raabymagle and Ladewing [26] found that box size exerted an influence on the time spent in sternal recumbence, which was significantly longer in box (2.5 × height at withers of the horse) than in smaller one (1.5 × height at withers of the horse). Stable structure has to be suitable in order to allow horses to interact with each other, when possible horses should live in social group. Clean, good quality and appropriate feed and bedding, fresh drinking water, and washing-down water must always be available.

To reduce air contaminants in the stable, an artificial ventilation system could be useful to guarantee sufficient air renewal to preserve the health and well-being of the animals as established by Council Directive 98/58/EC of 20 July 1998 concerning the protection of animals kept for farming purposes [27].

Monitoring environmental conditions and characterization of temperature humidity index, throughout the world used to generally characterize climate effect on animal performance, could be useful to reduce heat stress during race, training and housing.

Another fundamental element is represented by nutrition. In particular, intensive management feeding practice of sport horses is a basic matter that can influence welfare status. Some kinds of nutrition are represented by high energy diet, with reduced fibrous components and the supplement of meals twice a day. In this case, horses spend very little time feeding compared with free-ranging horses. This behavior occupies the majority of its time; it consists of locomotor activity as well as feeding [28]. Stable conditions, reducing this behavior [29] and favor the development of stereotypies [30]. Then, it is important that horses take up an important part of their energy in the form of roughage that has to be of high quality and dust free to reduce potential respiratory problems [31]. In addition, feeding practices have been associated with colic in horses. If meal size and composition have an effect on gastric emptying, this could be one of the mechanisms by which feeding practices are related to the occurrence of colic [32]. Ideally, changes in the type or amount of feed given a horse should be allowing the digestive system to adapt to different levels and physical forms of nutrients. Some feed changes can be made almost immediately, some require a few days, and others require a week or longer to assure a safe adjustment.

Training methods, training place and competition conditions must be taken into consideration also. Horses must only undergo training that matches their physical capabilities and level of maturity for their respective disciplines. They must not be subjected to any training methods that are abusive or cause fear or for which they have not been properly prepared. The animals should not be beaten or abused. Repeated or excessive force should not be used against the horse as a form of punishment. Basic education and handling of young animals are desirable, although strenuous training should be minimal to reduce the risks of injury and growth abnormalities. Training methods involving cruelty or repeated pain ‘insults’ should not be used.

Horses require regular exercise for a period of weeks before they are adequately conditioned for strenuous exercise. Significant experience and skill are required to be able to accurately judge the fitness of a horse and its ability to compete without causing it pain or distress.

Training place must have suitable and safe surfaces. All ground surfaces on which horses walk, train or compete must be designed and maintained to reduce the risk of injuries. Particular attention must be paid to the preparation, composition and upkeep of surfaces.

Competitions must not take place in extreme weather conditions if the welfare or safety of the horse may be compromised. Provision must be made for cooling horses quickly after competing in hot or humid conditions.

Guarantee a period of acclimation is necessary for horses that compete in environmental conditions different from the environment they are accustomed.

It could be useful, in conclusion, underline that horse and competitors must be fit, competent and in good health before they are allowed to compete and that participation in a competition of a horse clearly in state of distress or female in advanced stages of pregnancy is considered maltreatment. Abuse of drugs and medication that could be altering horse performance is a serious welfare issue and will not be tolerated. After any veterinary treatment, an adequate period has to pass before turn back in competition.

Abuse of a horse using artificial aids will not be tolerated, as well as the use of harnesses is likely to cause unnecessary suffering. The use of the whip (max 140 cm long) beyond reasonable limits is considered maltreatment, in particular, if the horse is whipped start boxes, after the arrival of the race, both on the track and the stables.

## Indirect Parameters

These parameters, even if subjective, since they are based on surveys carried out by the observer on the animal, they can provide a lot of useful elements to establish the rule of the welfare of the horse. They, in fact, as already mentioned, provide information on the environment and the living conditions of the animal through the relief of important elements on the animal itself, far from the places of rearing or training. These indicators could be divided into three categories: behavioral indicators, health-related indicators and descriptive indicators [25,33]. As behavioral indicators, the presence or the absence of tare and stereotyped behaviors, general attitude and response to human approach could be taking into consideration. The health-related records are: Body condition score (BCS), hair coat quality, capillary refill time (CRT), presence of lesions or scars on face and body, presence of respiratory (dyspnea, nasal discharge, coughing) or enteric (diarrhea) signs, gait, hoof horn quality, length and shape of the hoof, sole surface, state of mucous membranes, teeth and eyes.

Access to exercise, access to drinking water and company of other horses or other mammals can be considerate as descriptive indicators of good welfare [33].

The presence of good BCS, of a normal CRT, the absence of lesions and disease, a general good condition of the animal, so as the absence of stereotypic behavior, the presence of alert and of a friendly approach to observer are all elements suggestive of respect of good welfare. Furthermore others elements that must be underlined are the alteration of natural behavior and the loss of psycho - physical health when horse is socially isolated and subjected to exercise and drinking water restriction.

All these indicators can be correlated with each other, and a correlation can be found also between direct and indirect parameters. For example a bad quality in housing conditions (direct parameter) with poor air exchange rate could increase the levels of harmful substances as ammonia, allergen and CO_2_, causing the onset of respiratory signs (indirect parameter).

## Conclusions

The welfare of the trotter must never be subordinated to competitive or commercial influences. It is an essential condition for keeping horses that handlers, drivers, trainers, farriers and veterinarians have proper knowledge of the behavior, state of health and environmental conditions of the horses in order to fulfill their natural needs and guarantee the good welfare. This purpose could be obtained through observation and detection of specific status indicators of well-being in the horse. These parameters, in fact, taking into account the conditions of life of the animal and their impact on the psycho-physical state of the latter, can provide the basis for a proper management in order to obtain the respect of good welfare. Competent horse handlers should recognize the different behavioral patterns of animals and successful trainers should adapt their training methods to suit the particular equine. Abnormal physiological and behavioral responses to training and confinement should be recognized, and measures taken to correct them. In addition, it is necessary to prohibit any training methods that are abusive or cause fear or for which the horse has not been properly trained.

Persons responsible for the welfare of horses should be experienced or under the supervision of an experienced person and should acquire the maximum amount of knowledge and skill required to keep and handle equines. Often the wellbeing and usefulness of the equine will depend on the skill and the attitude of the individuals handling them. They should be able to recognize signs of ill-health from the lower change of behavior to the marked sign of pathology and should consult a veterinarian when necessary to diagnose and treat illness or injury.

Given the current lack of laws, we would like to recommend to the Legislator the adoption of clear and strict regulations to supervise and ensure horses welfare during training and competitions. These regulatory acts could be used by courts to determine whether particular actions or inactions are a breach of the animal welfare legislation.

## Authors’ Contributions

AP and GP generated the concept. CG and LP collected materials, draft and revised the manuscript. All authors read and approved the final manuscript.

## Competing Interests

The authors declare that they have no competing interests.
